# Advances in Understanding the Mechanism of Cap-Independent Cucurbit Aphid-Borne Yellows Virus Protein Synthesis

**DOI:** 10.3390/ijms242417598

**Published:** 2023-12-18

**Authors:** Verónica Truniger, Giuliano Sting Pechar, Miguel A. Aranda

**Affiliations:** Centro de Edafología y Biología Aplicada del Segura, Consejo Superior de Investigaciones Científicas (CEBAS-CSIC), 30100 Murcia, Spain; gspechar@cebas.csic.es (G.S.P.); m.aranda@cebas.csic.es (M.A.A.)

**Keywords:** cap-independent translation, polerovirus, CABYV, 3′-CITE, translation initiation enhancer, RNA-binding protein, eIF4A, eIF4G, HSP70

## Abstract

Non-canonical translation mechanisms have been described for many viral RNAs. In the case of several plant viruses, their protein synthesis is controlled by RNA elements in their genomic 3′-ends that are able to enhance cap-independent translation (3′-CITE). The proposed general mechanism of 3′-CITEs includes their binding to eukaryotic translation initiation factors (eIFs) that reach the 5′-end and AUG start codon through 5′-3′-UTR-interactions. It was previously shown that cucurbit aphid-borne yellows virus (CABYV) has a 3′-CITE, which varies in sequence and structure depending on the phylogenetic group to which the isolate belongs, possibly as a result of adaptation to the different geographical regions. In this work, the cap-independent translation mechanisms of two CABYV 3′-CITEs belonging to the Mediterranean (CMTE) and Asian (CXTE) groups, respectively, were studied. In vivo cap-independent translation assays show that these 3′-CITEs require the presence of the CABYV short genomic 5′-UTR with at least 40% adenines in cis and an accessible 5′-end for its activity. Additionally, they suggest that the eIF4E-independent CABYV 3′-CITE activities may not require either eIF4A or the eIF4F complex, but may depend on eIF4G and PABP. By pulling down host proteins using RNA baits containing both 5′- and 3′-CABYV-UTRs, 80 RNA binding proteins were identified. These interacted preferentially with either CMTE, CXTE, or both. One of these proteins, specifically interacting with the RNA containing CMTE, was HSP70.2. Preliminary results suggested that HSP70.2 may be involved in CMTE- but not CXTE-mediated cap-independent translation activity.

## 1. Introduction

The translation mechanisms of viral mRNAs were selected to allow them to recruit the translational machinery of the host while competing with host mRNAs and avoiding defense mechanisms that act at the translation level. Thus, only 20% of the known positive-sense single-stranded RNA viruses infecting plants have genomic and subgenomic RNAs that include the canonical 5′-cap structure and the 3′-poly(A) tail [1]. Some of them use their 5′- and/or 3′-termini in alternative gene expression strategies [2,3,4]. In several cases, functional RNA elements in their genomic 3′-ends that are able to control their cap-independent translation have been identified; these are grouped under the generic name of cap-independent translational enhancer elements or 3′-CITEs [2,4]. 3′-CITEs are modular transferrable RNA elements, and their acquisition can be associated with a selective advantage for the new recombinant virus [5]. Different classes of 3′-CITEs have been described that differ in sequence, structure, and translation-enhancing mechanisms [6,7]. 

The general mechanistic steps of 3′-CITEs involve the recruitment of eukaryotic translation initiation factors (eIFs) at the 3′-CITE and their delivery near the translation start site [6,7]. Different host eIFs have been shown to be involved in different 3′-CITE activities [6,7,8,9,10]. The barley yellow dwarf (BYDV) 3′CITE (BTE) was shown to bind eIF4F through its eIF4G subunit [11] that coordinates the interactions with eIF4A, eIF4B, and eIF4E [9]. On the other hand, the ISS 3′-CITEs from maize necrotic streak virus (MNeSV) and melon necrotic spot virus (MNSV) have been shown to bind eIF4F through its eIF4E subunit, with this binding being essential for their activities [12,13]. Also, the PTE 3′-CITE from pea enation mosaic virus 2 (PEMV2) has been shown to bind eIF4E with high affinity, even in the absence of eIF4G, although it needs the complete eIF4F complex for its activity [14]. The 3′-CITEs TED of satellite tobacco necrosis virus (STNV) and YSS from carnation Italian ringspot virus (CIRV) have been shown to bind wheat eIF4F and eIFiso4F, but with a preference for the former complex [15,16]. In contrast to the other 3′-CITEs, no eIFs that bind to the turnip crinkle virus (TCV) TSS have been described, but it was shown to directly recruit and bind to the P-site of the 60S subunit [17] and the 80S ribosome [18]. The two TSSs identified in PEMV2 have been shown to bind both to the 80S ribosome and the 60S and 40S ribosomal subunits [18,19]. 

The delivery of the eIFs bound to 3′-CITEs near the translation start site occurs through a 5′-3′-end interaction based on sequence complementarity in several cases [6,20]. In these cases, the presence of both genome ends is essential for the 3′-CITE-mediated cap-independent translation activity. Experimentally, this has been shown for the BTE of BYDV [21], the PTE of saguaro cactus virus (SCV) [22], the TED of pelargonium line pattern virus (PLPV) [23], the YSSs of CIRV [16,24] and tomato bushy stunt virus (TBSV) [25,26], and for the ISSs of MNeSV [13] and MNSV [20]. 5′-3′-interactions can also occur indirectly through ribosomes, as shown for TCV [27]. For the umbravirus PEMV2, which contains three 3′-CITEs, direct and indirect modes of interaction were proposed [28,29,30].

Cucurbit aphid-borne yellows virus (CABYV), a member of the polerovirus genus recently assigned to the *Solemoviridae* family [31], has a positive-sense RNA genome that lacks a 5′-cap and a 3′-poly(A)-tail [32,33]. Recently, different functional 3′-CITEs in the genomic 3′-UTRs of CABYV isolates belonging to different phylogenetic groups [34], the CXTE for the Asian, and the CMTE for the Mediterranean groups were identified [35]. The 3′-UTR sequences of Mediterranean isolates are nearly identical, and the 3′-UTR sequences of Asian isolates are also highly conserved. However, the first half of the 3′-UTRs, where the 3′-CITEs were identified, are quite different between both groups. These two 3′-CITEs, differing in sequence and structure and possibly acquired by recombination, evolved in different geographical regions, which may suggest that their respective 3′-CITEs are possibly better adapted to each region [35]. Notably, the translation of other polerovirus genomes was also proposed to be 3′-CITE-dependent. 

The aim of this study is to advance the knowledge of the molecular mechanism of CABYV protein translation in the host cell. This is of interest in basic research, but additionally, the identification of host proteins required for CABYV 3′-CITE activity can provide new targets for the generation of virus-resistant plants. The host factors required for the activity of the newly identified CABYV 3′-CITEs CXTE and CMTE are still unknown. Both are functional in melon plants silenced for eIF4E; thus, their activity must be eIF4E-independent [5,35]. In this manuscript, the molecular mechanisms of cap-independent translation initiation controlled by the CABYV 3′-CITEs CXTE and CMTE were studied, resulting in the identification of several host proteins that may be involved in their cap-independent translation activities. 

## 2. Results

### 2.1. The 5′-UTR Is Required for CABYV 3′-CITE Activities 

**Figure 1:**
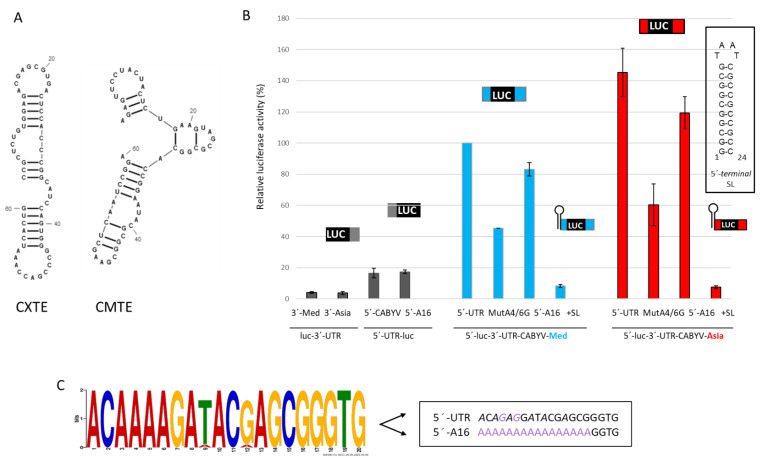
5′-UTR-dependent CABYV 3′-CITE activities. (**A**) Proposed secondary structures of CXTE and CMTE drawn with RNAcanvas [38]. (**B**) In vivo cap-independent translation efficiency of different luc constructs assayed in melon protoplasts. Vertical columns represent the luciferase activity measured (corresponding to the translation efficiency) relative to the activity obtained with the construct 5′-UTR-luc-3′-UTR of CABYV-Med (100%) [35]. Above the columns, a schematic drawing of the respective constructs assayed is shown (blue for CABYV-Sp sequences flanking luc, red for CABYV-Xin, gray for only one UTR). The stable SL-structure (∆G = −27.30 kcal/mol) that was added at the very end of the 5′-terminus (+SL) is shown to the right. Error bars are +/−SD of at least five independent experiments. (**C**) Consensus sequence of all CABYV 5′-UTR sequences available in Genbank shown as a stacked motif (https://meme-suite.org/meme/, accessed on 28 September 2023). The height of the letter shows the conservation of the nucleotide. On the right, the sequence of the mutated 5′-UTRs with A4 and A6 changed to G (MutA4/6G), marked in purple, and the first 16 nt exchanged with poly(A)_16_ (5′-A16) are boxed.

To study the 5′-UTR requirements of the CABYV CITEs, the firefly luciferase reporter gene was flanked with the genomic 5′- and/or 3′-UTRs of a Spanish isolate (Sq_04_1.9), representing the Mediterranean group and including the CMTE in its 3′-UTR, or with the 5′- and/or 3′-UTRs from the Xinjiang isolate, representing the Asian group and including the CXTE in its 3′-UTR [35]. Figure 1A shows the proposed secondary structures of both 3′-CITES as obtained after chemical structure probing in Miras [35]. Luciferase activities, corresponding to the in vivo cap-independent translation efficiencies, were measured after the expression of each RNA in melon protoplasts and represented relative to the activity obtained with the wild-type construct 5′-UTR-luc-3′-UTR of CABYV-Med (100%) (Figure 1B). Low translation efficiencies were determined for RNAs with only one UTR, 5′- or 3′-UTR, flanking the luciferase gene (Figure 1B, first four columns). The presence of both UTRs resulted in a 6.5 times higher translation efficiency for Mediterranean (fifth column) and 9.5 times higher translation efficiency for Asian UTRs (ninth column). These results confirmed that the 5′-UTRs are required in cis for the full CMTE and CXTE activities [35]. 

The very short CABYV 5′-UTRs (twenty nucleotides (nt) long) are identical between the Spanish and Xinjiang isolates, and only two nucleotides vary between all CABYV isolates: the first T is changed into A and the second G is also changed into A in three isolates [35], which results in a highly conserved consensus sequence (Figure 1C). Interestingly, these 5′-UTRs have a high purine content (75%): 8–10 adenines and 6–7 guanines out of 20 nt (Figure 1C). Thus, the possible role of these nucleotides in CABYV 3′-CITE activities was studied by lowering and increasing the A- and G-contents of the 5′-UTRs, respectively, by exchanging A4 and A6 for G. These mutations affected both 3′-CITE activities, reducing to 45% and to 38% those of CMTE and CXTE, respectively, compared to the wild-type activities (Figure 1B, sixth and tenth columns). Since the removal of A’s resulted in a reduction in the translation efficiency, the A content of the 5′-UTR was doubled by exchanging the first 16 nucleotides with 16 adenines, while maintaining its last 4 nucleotides. CMTE and CXTE were both functional with this poly(A)_16_ 5′-UTR (Figure 1B, seventh and eleventh columns), resulting in similar translation activities to the wild-type 5′-UTR (approx. 85%). The results of both experiments suggested that the adenines in the 5′-UTR are important for translation mediated by the CABYV 3′-CITEs.

The presence of a poly(A) leader sequence at the 5′-end of mRNAs has been shown to result in efficient translation initiation through its capacity to internally recruit the 40S ribosomal subunit [36], similar to internal ribosome entry sites (IRESs) known to control the translation of many viral proteins [37]. Thus, this possibility for CMTE and CXTE was analyzed. A stable stem-loop structure (SL), which had been previously shown to prevent cap-independent translation controlled by the MNSV-Ma5 3′-CITE (Ma5TE) [20], was added to the 5′-termini of the CABYV 5′-UTR-luc-3′-UTR constructs. These RNAs were poorly translated (Figure 1B, eight and twelfth column), suggesting that for the CMTE- and CXTE-mediated translation activities, the ribosome had to be able to enter the mRNA from its 5′-terminus, as previously shown for Ma5TE.

### 2.2. EIF4A Inhibition Does Not Affect CABYV 3′-CITE Activities

**Figure 2:**
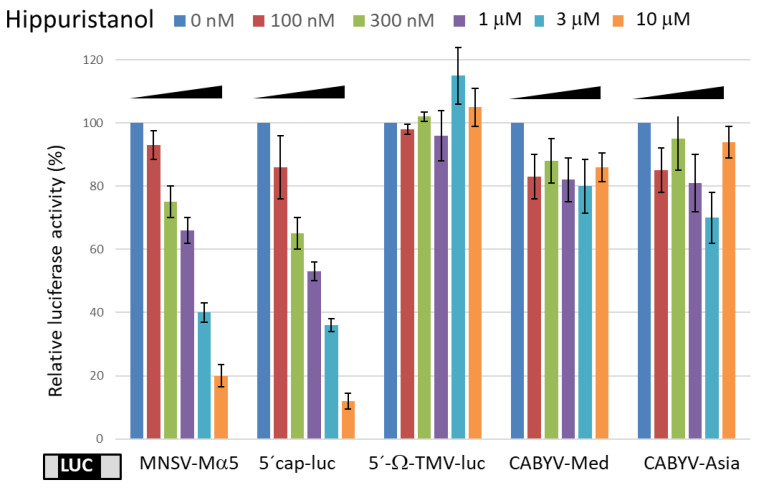
eIF4A-independence of CABYV 3′-CITE-mediated cap-independent translation. In vivo translation efficiency measured in protoplasts of different 5′-UTR-luc-3′-UTR constructs (bottom) in the presence of increasing concentrations of the eIF4A-inhibitor hippuristanol (0, 0.1, 0.3, 1, 3, 10 μM, as indicated at the top) relative to the activity in its absence (100%). Positive controls: eIF4A- and cap-dependent translation of luciferase gene flanked by plasmid sequence [8] and uncapped cap-independent eIF4A-dependent translation controlled by the Ma5TE [20]. Negative control: uncapped eIF4A-independent [41] translation controlled by the Ω element of TMV (Ω-luc-poly(A)_60_), kindly obtained from A. Miller [43]. Error bars are +/−SD of at least five independent experiments.

Several functional inhibitors of translation initiation factors in animal cells have been described, but these have seldom been tested in plant cells. In the case of the eIF4A inhibitor hippuristanol [39], this has been shown to inhibit eIF4A activity in wheat cells, apart from animal cells [40]. To learn if the cap-independent CABYV 3′-CITE-mediated translation depends on eIF4A, the effect of adding increasing concentrations of hippuristanol on the in vivo translation efficiency of the CABYV 5′-UTR-luc-3′-UTR constructs in melon protoplasts was tested. The results showed that increasing concentrations of hippuristanol reduced the translation efficiencies of luc constructs flanked by the MNSV-Mα5 UTRs, previously shown to depend on eIF4A [20], as well as of a capped RNA construct with a plasmid sequence flanking the luc gene [8] (Figure 2). On the other hand, the translation efficiencies mediated by the CMTE and CXTE were not affected. Similarly, the tobacco mosaic virus (TMV) Ω-element-mediated cap-independent translation, known to be independent of eIF4A [41,42], was not affected (Figure 2). These results suggested that CABYV 3′-CITE-mediated cap-independent translation may be eIF4A-independent.

**Figure 3:**
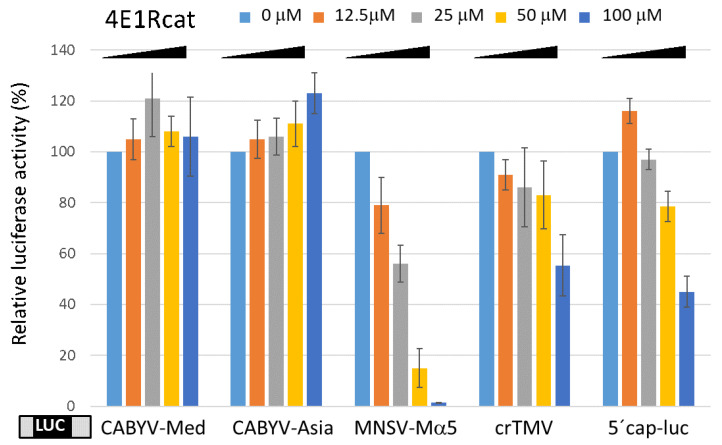
Effect of inhibition of eIF4F complex formation on translation efficiency. In vitro translation efficiency in wheat germ extract of CABYV uncapped 5′-UTR-luc-3′-UTR and capped control constructs. The *Y*-axis shows relative translation activity in absence (0 μM = 100%) and in presence of increasing concentrations of 4E1Rcat (0–100 μM, as indicated below), which competes against eIF4G for eIF4E binding. RNA of uncapped luc construct flanked by both MNSV-Mα5 UTRs, whose translation controlled by the Ma5TE is eIF4F-dependent [20], serves as the positive control. 5′cap-luc: capped RNA construct of luciferase gene flanked by plasmid sequence [8]; crTMV: *luc* gene flanked by crTMV IRES at the 5′-end and its 3′-UTR at the opposite end [43]. Error bars are +/−SD of at least five independent experiments.

The effect of the commercially available inhibitor 4E1Rcat, which blocks the assembly of the eIF4F complex and disrupts preformed eIF4F by competing against eIF4G for eIF4E binding, was also tested. The addition of increasing concentrations (up to 100 μM) of 4E1Rcat to wheat germ extract strongly reduced the cap-independent translation of a luc construct flanked by the MNSV-Ma5 5′-and 3′-UTRs (Figure 3). The Ma5TE has been shown to require eIF4F for activity [12], thus serving as the positive control. The cap-dependent translation of the capped luc construct was less affected, but a progressive reduction upon the addition of increasing 4E1Rcat concentrations could be observed (Figure 3). On the other hand, in agreement with the finding that IRESs promote cap- and eIF4E-independent translation [37], the translation of a luc construct containing the crTMV-IRES (crucifer TMV) at its 5′-end [43] was nearly unaffected (only at the highest 4E1Rcat concentration). Interestingly, the translation efficiency mediated by the CABYV CITEs was not affected by the addition of 4E1Rcat (Figure 3), suggesting that their activity is independent of the eIF4F complex. This result is in agreement with the eIF4E-independence of the CABYV 3′-CITEs [35].

### 2.3. Arabidopsis Proteins Involved in CABYV 3′-CITE-Mediated Translation Activity

**Figure 4:**
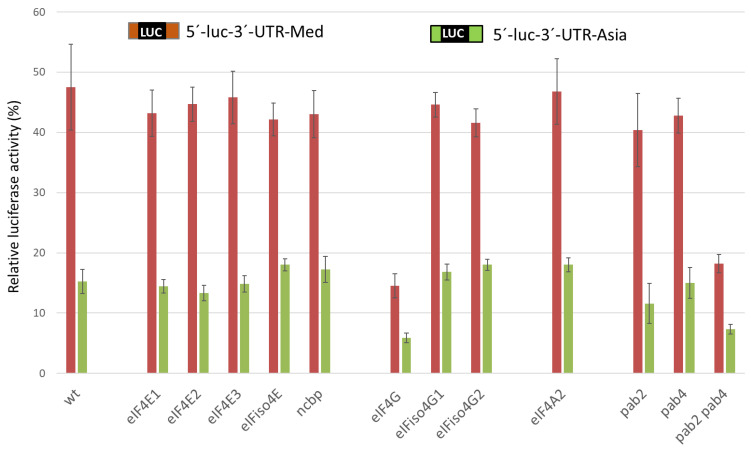
CABYV 3′-CITE-mediated cap-independent translation in *Arabidopsis* lines mutated in eIFs. In vivo translation efficiency of CABYV 5′-UTR-luc-3′-UTR constructs (including CMTE, Med; or CXTE, Asia) relative to the activity of a capped control construct (100%). Nottingham *Arabidopsis* Stock Centre (NASC) *Arabidopsis* homozygous knockout mutant lines in *eIF4E1* (*cum1-1* = N6552 and N656042), *eIF4E2* (N663501), *eIF4E3* (N663174), *eIFiso4E* (N673222), *ncbp* (N679102), *eIF4G* (*cum2-1* = N6553 and N676550), *eIFiso4G1* (N673021), *eIFiso4G2* (N654727), *eIF4A2* (N662446), *pab2* (N8174), *pab4* (N8175), and *pab2pab4* (N8177). All mutant lines, with the exception of the EMS point mutants in *cum1-1* and *cum2-1* [46], are T-DNA insertion lines [47]. Error bars are +/−SD of at least five independent experiments.

*Arabidopsis* has been described as a host for CABYV [33,44]. We took advantage of the availability of *Arabidopsis* lines mutated in genes coding eIFs in the Nottingham *Arabidopsis* Stock Centre. The cap-independent translation efficiency of CABYV 5′-UTR-luc-3′-UTR RNAs relative to the cap-dependent translation (100%) in protoplasts of *Arabidopsis* homozygous mutant lines was analyzed. Fourteen mutant lines were tested, with knockouts in *eIF4E1* (two different lines), *eIF4E2*, *eIF4E3*, *eIFiso4E*, *ncbp*, *eIF4G* (two different lines), *eIFiso4G1*, *eIFiso4G2*, *eIF4A2*, *pab2*, *pab4*, and the double-mutant *pab2pab4*. In the protoplasts of *Arabidopsis* mutated in the different *eIF4E* isoforms (Figure 4, second to sixth pairs of columns), the cap-independent translation efficiencies mediated by the CABYV CITEs were similar to those in wild-type *Arabidopsis* (Figure 4, first pair of columns). Also, the translation efficiencies in the *eIF4A2*, *eIFiso4G1*, and *eIFiso4G2* mutants were not affected (Figure 4, eighth to tenth pairs of columns). Contrastingly, the translation efficiencies of RNAs controlled by each of the CABYV CITEs were reduced nearly 3-fold in protoplasts of the *eIF4G* mutant when compared to their activities in wild-type *Arabidopsis* (Figure 4, seventh pair of columns). On the other hand, translation efficiencies were similar to wild-type in the poly(A)-binding protein (PABP) mutant lines *pab2* and *pab4* (Figure 4, eleventh and twelfth pairs of columns), but a reduction could be observed in the double-mutant *pab2pab4* (Figure 4, last pair of columns). In *Arabidopsis*, a potential functional redundancy exists for PABP, as eight isoforms (*pab1-8*) have been identified [45]. These results suggest that eIF4G and the PABPs 2/4 may be required for CMTE and CXTE activity.

Competition for eIF4G protein was studied by the in trans addition of increasing concentrations of BTE-RNA (BYDV 3′-CITE), which has been shown to bind eIF4G [11]. The 5′-luc 3′-UTR CABYV RNAs were translated in wheat germ extract. Increasing concentrations of eIF4G-binding BTE were added. The CABYV 3′-CITE-mediated translation efficiency was inhibited in the presence of increasing concentrations of competitor BTE (Supplementary Figure S1). This result supports that eIF4G may be important for CMTE- and CXTE-mediated activity.

### 2.4. Capture of Melon Proteins Binding to the CABYV 3′-CITEs

**Figure 5:**
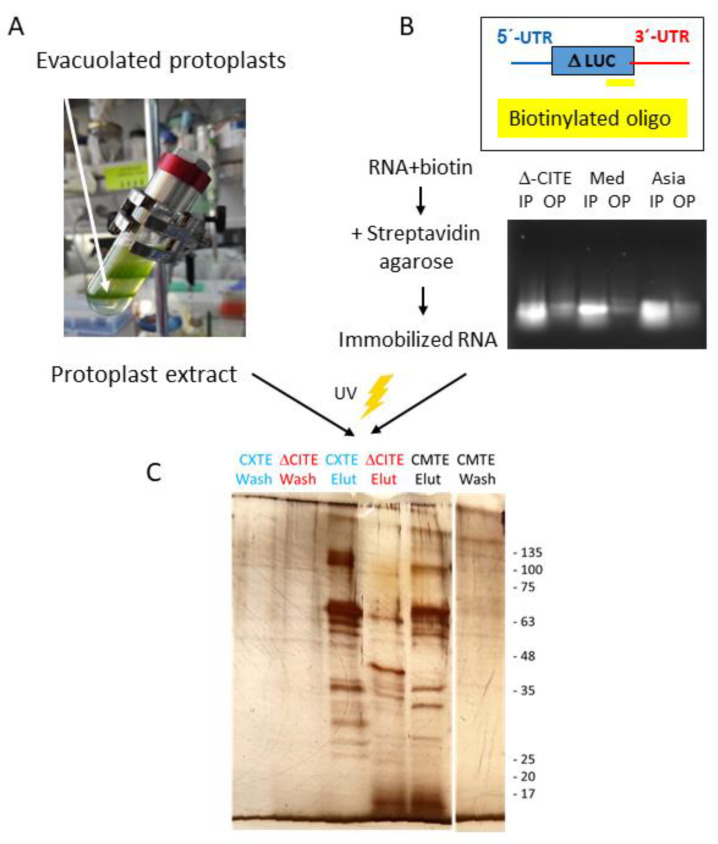
Pull-down of RBPs. (**A**) Percoll gradient performed to purify evacuolated melon protoplasts that float in the 60–40% interphase (lower ring, marked with white arrow). (**B**) Scheme of the RNA constructs and the biotinylated complementary oligonucleotide hybridized to it. The agarose gel shows RNA before (input—IP) and after (output—OP, supernatant) incubation with streptavidin agarose. (**C**) Silver-stained SDS-PAGE showing the last wash and the elution steps of the three RNAs studied, containing either a CMTE, a CXTE, or no 3′-CITE (∆-CITE) in its 3′-UTR.

With the aim of delving into the mechanisms of both CABYV 3′-CITEs, melon proteins interacting specifically with CXTE and/or CMTE were identified. An RNA-centric method in which a selected RNA hybridized to a biotinylated complementary oligonucleotide was immobilized on streptavidin–agarose beads was developed. In this way, the proteins from an extract from evacuolated melon protoplasts interacting with these RNAs could be captured and identified by mass spectrometry (MS). An important problem while working with RNA together with plant extracts is the high content of RNases in plant cells. Since RNases and proteases are mainly present in the vacuoles of protoplasts, these could be avoided by the purification of evacuolated protoplasts (Figure 5A).

The RNAs used in this experiment should preferably be translatable, but the CABYV 5′-UTR-luc-3′-UTR constructs used in the previous experiments were too large (over 2000 nt), resulting in inefficient binding to the streptavidin agarose beads. Thus, part of the luc-coding sequence was deleted so that 102 nt of the luc gene (coding for 33 amino acids) followed the CABYV 5′-UTR. The truncated luc gene was followed either by the 3′-UTR of Mediterranean or Asian CABYV 3′-UTR or the Mediterranean 3′-UTR lacking its CMTE (∆-CITE). This last construct allowed discriminating between RBPs binding to the RNA in the absence of the 3′-CITE and those binding specifically to CMTE or CXTE. Depending on the length of the 3′-end of the construct, its final size was between 225 and 285 nts. To preserve the secondary structure of the UTRs in the RNA constructs, the biotinylated oligonucleotide was complementary to 30 nt at the 3′-end of the truncated *luc* gene. After the hybridization step, the RNAs bound efficiently to the streptavidin agarose beads. The near absence of RNA in the supernatant of the agarose + RNA mix confirmed efficient RNA immobilization (Figure 5B). Subsequently, the immobilized RNAs were incubated with an extract of evacuolated melon protoplasts. Bach-Pages and colleagues [48] showed the importance of fixing the RBP to the RNA; thus, the RBPs were UV-crosslinked to the RNAs. UV light is able to crosslink nucleic acid to protein at zero distance and does not crosslink protein to protein. After loading this mixture on a spin column and performing six wash steps, the RNA-RBP complexes were eluted via the application of heat, resulting in the disruption of the hydrogen bonds between the RNA and the biotinylated oligonucleotide. Wash and elution steps were controlled by loading samples on SDS-PAGE followed by silver staining (Figure 5C).

**Figure 6:**
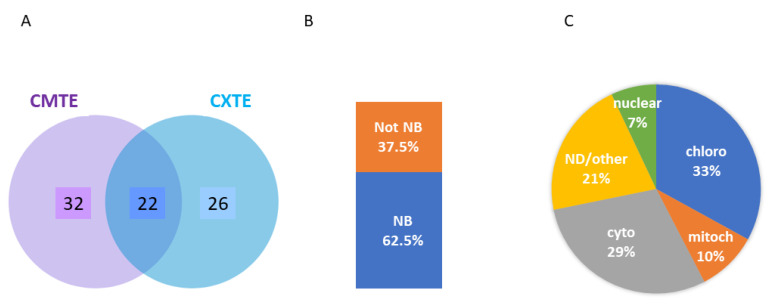
Analysis of proteins binding preferentially to CABYV 3′-CITEs. (**A**) Venn diagram (http://www.ehbio.com/test/venn/#/, accessed on 30 September 2023) of the 80 proteins pulled down with a 1.9-fold or higher abundance with the RNA constructs containing the CMTE or CXTE than with the ∆-CITE RNA (normalized abundances; percentage of identified protein relative to the average of abundance of that protein in all samples). (**B**) Percentage of the identified proteins annotated as “nucleotide binding” (NB) in the molecular function GO term. (**C**) Localization of these proteins as annotated in the cellular component GO term: chloro—chloroplastic, mitoch—mitochondrial, nuclear, cyto—cytosolic and ND/other—not defined or other localization.

Among the proteins identified by LC-ESI-MS/MS, those that were found to bind to the CMTE and/or CXTE RNAs more frequently than to the ∆-CITE RNA were selected (>1.9-fold higher, 80 proteins). Twenty-two proteins bound with similar efficiency to both 3′-CITEs, while 32 and 26 showed preferences for the CMTE and CXTE, respectively (Figure 6A). A GO term analysis showed that 62.5% are described as nucleotide-binding proteins for the GO term molecular function (Figure 6B); for the GO term biological process, 19% are proteins involved in translation, 24% in photosynthesis, 8% are heat shock proteins (HSP), and 4% histones. For the GO term cellular component, 29% had a cytosolic, 33% a chloroplastic, 10% a mitochondrial, and 7% a nuclear localization, while for 21%, the localization was different or not defined (Figure 6C).

**Figure 7:**
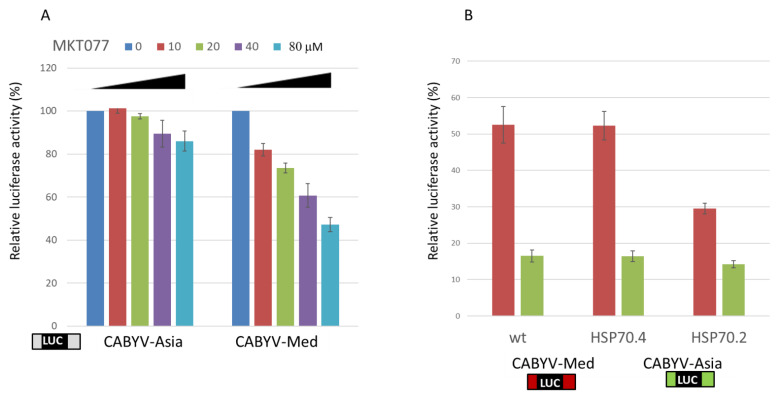
Possible role of HSP70.2 in CABYV 3′-CITE-mediated cap-independent translation. (**A**) In vivo translation efficiency of 5′-UTR-luc-3′-UTR constructs flanked with Mediterranean or Asian CABYV-UTRs (bottom) measured in protoplasts in the presence of increasing concentrations of the HSP70 inhibitor MKT077 (10, 20, 40, 80 μM, as indicated at the top) relative to the activity in its absence (0 μM, 100%). (**B**) In vivo translation efficiency of CABYV 5′-UTR-luc-3′-UTR constructs (including CMTE, Med or CXTE, Asia) relative to the activity of a capped control construct (100%). NASC *Arabidopsis* homozygous knockout mutant lines in *hsp70.2* (N663161) and *hsp70.4* (N655706). Error bars are +/−SD of at least four independent experiments.

The heat shock protein (HSP) 70.2 and HSP 70.2-like proteins were differentially pulled down with the two CABYV CITEs, being identified 5 and 10 times, respectively, more frequently using the RNA including the CABYV-Med UTRs than using the RNAs including the CABYV-Asia UTRs or the ones with the 3′-CITE deletion. Thus, a possible role for HSP70 in the CMTE-mediated protein translation mechanism was studied. First, the effect of MKT077, an inhibitor of HSP70 shown to function in plant cells [49], on in vivo cap-independent translation through its addition to melon protoplasts that were electroporated with the luc constructs was analyzed. Increasing the concentration of MKT077 more severely affected CMTE- than CXTE-mediated translation (Figure 7A). We also compared the translation efficiencies of the two uncapped CABYV CITEs containing RNAs relative to a capped construct in *Arabidopsis* protoplasts mutated in *hsp70.2*, *hsp70.4,* or the wild type (Figure 7B). The translation efficiencies for both constructs were similar in the hsp70.4 mutant and wild-type *Arabidopsis* protoplasts. On the other hand, the translation efficiency of the CABYV-Asia construct was not affected in comparison to the wild type in *Arabidopsis* protoplasts of the hsp70.2 mutant, although the translation efficiency of the CABYV-Med construct was reduced 1.8-fold (Figure 7B). These results suggested that HPS70.2 could play a role in the CMTE-mediated cap-independent translation mechanism.

## 3. Discussion

The work presented here provides a significant advancement in the understanding of the mechanisms of the cap-independent 5′-UTR-dependent translation enhancers of CABYV, CMTE, and CXTE. The results presented suggest that the ribosomes need to enter and start mRNA scanning from the very 5′-terminus of the CABYV genome for the activity of both CABYV 3′-CITEs. The presence of at least 40% of adenines in the sequence of the CABYV’s very short genomic 5′-UTR was also shown to be important for 3′-CITE activity.

Ribosome scanning from the 5′-end of the viral genome has been shown in several cases of 3′-CITE-mediated translation, for instance, for BTE [21] and Ma5TE [20]: the addition of a sequence stretch that folds into a stable SL at the 5′-termini inhibited BTE- and Ma5TE-mediated translation. For cap-dependent translation, the presence of stable SL structures (∆G = −18 kcal/mol) downstream of the mRNAs start codon has also been shown to block translation [50]. Here, it is shown that the presence of a stable SL at the 5′-terminus also avoids both CABYV 3′-CITE-mediated translation, suggesting that their activity requires ribosome scanning from the 5′-terminus. Additionally, in the case of several 3′-CITEs, circularization of the viral RNA has been shown to occur through a 5′-3′-interaction based on sequence complementarity [7]. This circularization is expected to bring the eIFs bound to the 3′-CITE near to the start codon. No 5′-3′-end complementary sequence stretches involved in translation mediated by CMTE or CXTE in CABYV could be identified. Thus, circularization of the CABYV genomic RNA may occur through another mechanism.

The results presented here suggest that the high content of adenines in the 5′-UTR sequence (40–50%) is important for efficient CMTE- and CXTE-mediated translation, as the reduction in the A-content led to reduced translation efficiencies, while the substitution of the first 16 nucleotides with poly(A)_16_ did not negatively affect translation. Interestingly, poly(A) leaders of 8–12 residues present in poxvirus 5′-UTRs have been shown to boost virus protein production [51,52]. Also, Xu and colleagues [53] found that the 5′-leader sequences of transcripts, preferentially translated in response to pattern-triggered immunity, were highly enriched in purines and included a consensus motif of 15 nucleotides named the R-motif. The R-motif has been recently identified in plants forming part of a mechanism of pathogen-induced cap-independent translation [54]. R-motif-dependent translation has been shown to function by internal ribosome entry, following its direct binding to PABP and promoting eIFiso4G-mediated translation [53,54,55]. The CABYV 5′-UTR sequence has high similarity with the R-motif (Supplementary Figure S2). Thus, on the one hand, the possibility that the CABYV 5′-UTR functions similar to an R-motif would explain the difference in the translation efficiencies observed between the 5′-UTR-luc and the luc-3′-UTR RNAs shown in Figure 1, which are about 4-fold higher for the 5′-UTR-luc and 5′A16-luc constructs. On the other hand, the results show that efficient cap-independent translation depends on an accessible 5′-terminus, excluding the possibility of internal ribosome entry. Additionally, the CABYV 5′-UTR acts synergistically together with its 3′-UTR in translation. In any case, one possible role for the A-rich sequence could be the direct binding of PABP to the 5′-UTR as part of the CABYV 3′-CITE-mediated translation mechanisms. The result of the reduction in the CABYV 3′-CITE-mediated translation efficiencies in the *Arabidopsis* double-mutant line pab2pab4 suggests that PABP could indeed be involved.

CABYV 3′-CITE-mediated cap-independent translation was previously shown to be eIF4E-independent in melon [35]. The translation experiments with protoplasts of *Arabidopsis* mutants confirmed this but suggested that eIF4G could be important for the CMTE and CXTE activities. This is supported by the sensitivity of CABYV 3′-CITE-mediated translation to the presence of the eIF4G-binding BYDV-3′-CITE BTE. Interestingly, Terenin and colleagues (2013) found that eIF4G could bind to a poly(A) rich sequence, UAAAAA, introduced into the 5′-UTR of mRNAs, making their translation less dependent on the cap [56]. This translation mechanism was dependent on an accessible 5′-terminus. These findings are in line with the results showing that translation mediated by the CABYV 3′-CITEs depends on an accessible 5′-terminus. Thus, another possible role for the A-rich CABYV 5′-UTR sequence could be the binding of eIF4G. However, considering that the general mechanism proposed for 3′-CITEs suggests its direct binding to eIFs (eIF4E/G/F), another possibility would be that eIF4G binds to the CABYV 3′-CITEs. In that case, circularization of the RNA could be achieved through the protein–protein interactions between eIF4G and the PABP bound to the A-rich 5′-UTR. This eIF4G/PABP interaction would position the translation initiation complex near the start codon. Further experiments are required to test these models.

The results obtained with the *Arabidopsis* eIF-mutants encourage to generate melon mutants edited in *eIF4G*. Unfortunately, although melon plants edited in *eIF4G* by CRIPSR/Cas 9 technology were obtained, it was impossible to maintain this mutation in homozygosis, leading to eIF4G knockout, due to the occurrence of a lethal phenotype. Other groups working with rice came to the same conclusion [57,58]. Thus, it was not possible to confirm if eIF4G is a possible pro-viral/susceptibility factor for CABYV.

With respect to other eIFs, the in vivo translation experiments in the presence of hippuristanol allow the suggestion that CABYV 3′-CITE-mediated translation does not require eIF4A. The requirement for eIF4A in translation has been shown to be in direct proportion to the degree of 5′-end secondary structure of the mRNA, as it is supposed to unwind it, facilitating ribosome entry [59]. The fact that the CABYV 5′-UTR is very short, without a secondary structure, supports the possible eIF4A-independence of CABYV protein synthesis.

A total of 80 proteins that specifically interacted with the CABYV 3′-CITEs were identified through RBP pull-down experiments. While 62.5% of these are annotated as nucleotide-binding proteins, 37.5% are not, including proteins involved in photosynthesis and metabolic enzymes. These percentages are similar to the ones described for the mRNA-binding proteome from *Arabidopsis*, ranging from 60 to 75% [48,60]. With regard to the annotated localization of the identified RBPs, the distribution was similar when comparing the data obtained here with the mRNA-binding proteome described by Bach-Pages and colleagues [48]: 33% vs. 34% are localized to chloroplasts, 29% vs. 33% to the cytoplasm, and 10% vs. 9% to mitochondria. The data obtained here differed in the RBPs localized to the nucleus, with 7% vs. 24%; there were significantly fewer in the case described here. When comparing the annotations of the identified RBPs in the GO term biological process, between ours and the mRNA-binding proteome published by Zhang and colleagues [60], it should be emphasized that similar percentages of proteins involved in photosynthesis were identified in this study (approx. 24%), although fewer histones (4% instead of 18%) and many more heat shock proteins (8% instead of 1.4%) and proteins involved in translation (19% instead of 2%) were identified. All these differences can be explained with the different specificities of the RNAs used for RBP pull-downs, that in this study were specific RNAs with viral UTRs, while in Zhang and colleagues’ study [60] were mRNAs containing a poly(A)-tail. From the 80 RBPs identified here, 22 were able to interact with both CABYV 3′-CITEs, including, among others, actin, shown to be important for viral intracellular movement by RNA anchoring [61]; oxygen-evolving enhancer proteins, involved in plant antiviral defense [62]; and histones, important in defense response to virus infection [63]. To our surprise, we could not identify reliably any eukaryotic translation initiation factors in our pull-down experiments. Maybe the reason for this is that the translation initiation complex could not be fixed because we had decided that the RNAs should be translatable. Regarding eIF4G, this protein was described as the least abundant among the eIF4 factors [64].

From the proteins that were preferentially pulled down with one of the CAVYV 3′CITE-RNAs, HSP70.2 caught our attention, as it was pulled down more than five times more frequently with the CMTE-containing RNA. The experiments performed to further study a possible role of HSP70.2 in CMTE-mediated translation supported this result since, in melon protoplasts, the HSP70-inhibitor MKT077 reduced cap-independent CMTE-mediated translation to a greater extent than CXTE-mediated translation, and in protoplasts of the *Arabidopsis* hsp70.2 mutant line, the CMTE-mediated translation efficiency was reduced, while the CXTE-mediated translation was similar to that in wild-type *Arabidopsis*. Thus, HSP70.2 may be involved in the CMTE-mediated translation mechanism. HSP70s have been described to have RNA-binding and mRNA-stabilizing functions independent of their chaperone function [65]. Also, the multiplication of different viruses has been shown to depend on HSP70 [66], often playing a role in virus replication [67]. For the plant tombusvirus TBSV, *Arabidopsis* HSP70.2 has been shown to have pro-viral functions, stimulating its replication [49]. Additionally, in several cases, a direct role of HSP70 in virus translation has also been described. The knockdown of *hsp70* was shown to reduce IRES-mediated Hepatitis C virus, echovirus, encephalomyocarditis virus, and enterovirus A71 translation [68,69,70]; HSP70 was also shown to promote coxsackievirus protein translation [71]. Notably, another member of the heat shock protein family, HSP101, was shown to bind to the Ω element of the plant virus TMV, enhancing its activity in virus protein translation [72,73]. Further experiments are needed to confirm the possible role of HSP70.2 in the CMTE-mediated translation of CABYV viral proteins.

## 4. Materials and Methods

### 4.1. RNA Constructs

The RNA constructs analyzed here with the 5′- and or 3′-UTR of CABYV flanking the firefly luciferase (luc) gene were previously described [35]. The insertion of a stable SL at the 5′-end (see Figure 1), exchange of the first 16 nt of the CABYV 5′-UTR with poly(A)_16_, and mutation of A4 and A6 to G4 and G6 were obtained by amplification of the whole plasmid with complementary primers flanking these sequences on both sides; subsequent DpnI digestion to digest the bacteria-derived input plasmid and the selection of the mutant plasmids [74]. The partial deletion of the luc gene (∆-luc) was also achieved by in vitro mutagenesis. The RNAs synthesized from these ∆-luc constructs were used in the pull-down experiments described below. All new plasmid constructs were sequenced. These luc constructs were amplified by PCR with the high-fidelity Prime Star HS DNA polymerase (Takara, Kusatsu, Japan) and transcribed in vitro (MEGAscript; Thermo Fisher Scientific, Waltham, MA, USA). The capped construct used here corresponded to the luc gene flanked at both ends with plasmid sequence [35].

### 4.2. In Vivo and In Vitro Translation Assays

*Arabidopsis* protoplasts were prepared from leaves according to the protocol by Yoo [75]. The protoplasts were prepared from young melon cotyledons (8 days after seed planting), following the previously published protocol [8]. The electroporation of protoplasts with in vitro-synthesized RNA for in vivo translation studies was performed in the same manner for *Arabidopsis* or melon protoplasts, following [35,76].

In vitro translation assays with wheat germ extract (WGE, Promega Corp., Madison, WI, USA) were performed by incubating 2 pmol of RNA for 2 h at 25 °C in a final volume of 20 μL following the WGE instructions.

### 4.3. Purification of Evacuolated Protoplasts and Extract Preparation

Protoplasts from melon cotyledons were isolated following [8]. Evacuolated protoplasts were obtained by percoll gradient centrifugation (GE Healthcare, Chicago, USA) [77]. The gradient was obtained by overlaying 4 mL of each solution, starting with 60% percoll at the bottom, followed by 40, 30, and 15% percoll solutions (each in 0.6 M Mannitol, 20 mM MgCl_2_, 8 mM HEPES KOH pH 7.6). The protoplasts were carefully pipetted on the top. Centrifugation conditions were 10,000× *g*/25 °C/1 h (fixed-angle rotor). Vacuolated protoplasts were floating in the 60–40% interphase (lower ring), while protoplasts with vacuoles formed a second ring floating above (Figure 5A). The vacuolated protoplasts were recovered by puncturing the tube at its bottom, diluted in a 5-fold volume with 0.5 M Mannitol pH 5.7 and precipitated by centrifugation (150 g/10 min). The pellet was resuspended in 1–2 mL of buffer B 2× (30 mM HEPES-KOH pH 7.8; 100 mM KAc; 4 mM MgAc; 2 mM EDTA; 10% glycerol) with an additional 2 mM DTT and lysed in ice by sonication for 3 min (30 pulses of 3 s followed by 3 s rest). The extract obtained was centrifuged at 1000× *g* for 10 min at 4 °C and frozen in aliquots at −80 °C. Absence of RNase activity in the extract was confirmed by observation of the maintenance of integrity of RNA, after its incubation with the extract for 15 min at 37 °C, followed by agarose gel electrophoresis and RNA staining with Redsafe.

### 4.4. Capture of RNA-Binding Proteins from Vacuolated Protoplast Extracts: RNA Centric Method

Equimolar amounts of in vitro-transcribed RNA and biotinylated complementary oligonucleotide (5′-biotin-TCTCTCTGATTTTTCTTGCGTCGAGTTTT-3′) were hybridized (in buffer B 1×) by slowly cooling down from 55 to 25 °C. An amount of 50 μL of streptavidin agarose (High-capacity streptavidin agarose resin Thermo Scientific, Waltham, MA, USA) was washed twice with 150 μL of buffer B 1× by centrifugation at 200× *g* for 30 s. Then, 200 pmol of RNA hybridized to biotinylated oligonucleotide was allowed to bind to the precipitated streptavidin agarose beads by incubation at 20 °C with occasional mixing for 30 min. Binding was confirmed by loading 1 μL of input RNA mix and 1 μL of supernatant after incubation (output) on an agarose gel (Figure 5B), followed by electrophoresis and Redsafe staining.

To reduce unspecific binding, the extract from vacuolated protoplasts (200 μL) was pre-incubated for 1 h (5 °C, rotation) with streptavidin agarose (50 μL, without bound RNA). After this pre-incubation, the unbound extract (200 g/30 s) was first supplemented with 160 units of RNAse inhibitor (NZYtech, Lisboa, Portugal) and 20 μg/mL tRNA (Sigma, St. Louis, MI, USA), and then mixed with the RNA previously bound to the streptavidin agarose followed by incubation at 5 °C with rotation for 2 h. The mixture was then distributed into 20 μL drops on parafilm and exposed to UV light (150 mJ/cm^2^) in a crosslinker (distance to lamp 12 cm) [60]. UV light crosslinks nucleic acid to protein at zero distance and has an advantage over other crosslinking methods in that it does not crosslink protein to protein, but the efficiency of crosslinking is low [48]. Several doses of UV light treatments were tested, but above 150 mJ/cm^2^, a reduction in the translatability was observed in the RNAs, which were possibly damaged by the UV light. The same dose was used in [60]. The drops were collected and loaded onto a spin column (Chromotek, Planegg, Germany) for the wash steps (100 g 15 s) with 2× 150 μL of buffer B 2× supplemented with 2 mM DTT and 20 μg/mL tRNA, followed by 2× 150 μL of buffer B 2× supplemented with 100 mM LiCl, 2 mM DTT, and 20 μg/mL tRNA; the fifth wash step was performed with 150 μL of buffer B 1× supplemented with 2 mM DTT and 20 μg/mL tRNA, and the sixth/last step was performed with 60 μL of buffer B 1×. RBPs were eluted by hydrogen bond disruption between the biotinylated oligonucleotide and the RNA through the application of heat, which resulted in the separation of the RNA from the streptavidin agarose beads. For this step, 60 μL of buffer B 1× pre-heated at 55 °C was loaded onto the column followed by incubation for 5 min at 55 °C and subsequent centrifugation for elution. For visualization, the wash and elution steps were loaded on SDS-PAGE gels followed by silver staining (Figure 5C, Pierce, Thermofisher). Eluted RBP proteins were determined by LC-ESI-MS/MS (medium gradient) (https://www.cnb.csic.es/index.php/es/investigacion/servicios-cientificos/proteomica, accessed on 1 February 2022). The identified melon proteins were inferred from homology (Uniprot database; https://www.uniprot.org/uniprotkb/?query=*, accessed on 1 February 2022).

## 5. Conclusions

It can be concluded that CABYV 3′-CITE-mediated cap-independent translation is 5′-UTR-dependent, with its high content of adenines and an accessible 5′-terminus being important for this activity. The mechanism may be eIF4A- and eIF4F-independent but dependent on eIF4G and PABP. It can be proposed that either eIF4G or PABP bind directly to the adenine-rich 5′-UTR sequence for CABYV 3′CITE-mediated translation. If PABP would bind to the 5′-UTR, eIF4G could bind to the 3′-CITE, and RNA circularization would be achieved by eIF4G/PABP protein–protein interaction. In RNA:RBP pull-down experiments using RNA constructs containing both 5′- and 3′-CABYV-UTRs, 80 RBPs were identified. These RBPs were pulled down either with both 3′-UTRs (Mediterranean or Asian), or specifically with either the Mediterranean or the Asian RNA constructs. Among these, HSP70.2 was preferentially pulled down with CMTE, and the preliminary results presented allow the suggestion that HSP70.2 could be involved in CMTE- but not CXTE-mediated cap-independent translation activity.

## Data Availability

Data are contained within the article and Supplementary Materials.

## Supplementary Materials

The supporting information can be downloaded at: https://www.mdpi.com/article/10.3390/ijms242417598/s1.
