# Supplementary figures and images for: Advances in Understanding the Mechanism of Cap-Independent Cucurbit Aphid-Borne Yellows Virus Protein Synthesis
Source: Int J Mol Sci. 2023 Dec 18;24(24):17598. doi: 10.3390/ijms242417598 (PMC10744285; doi:10.3390/ijms242417598)

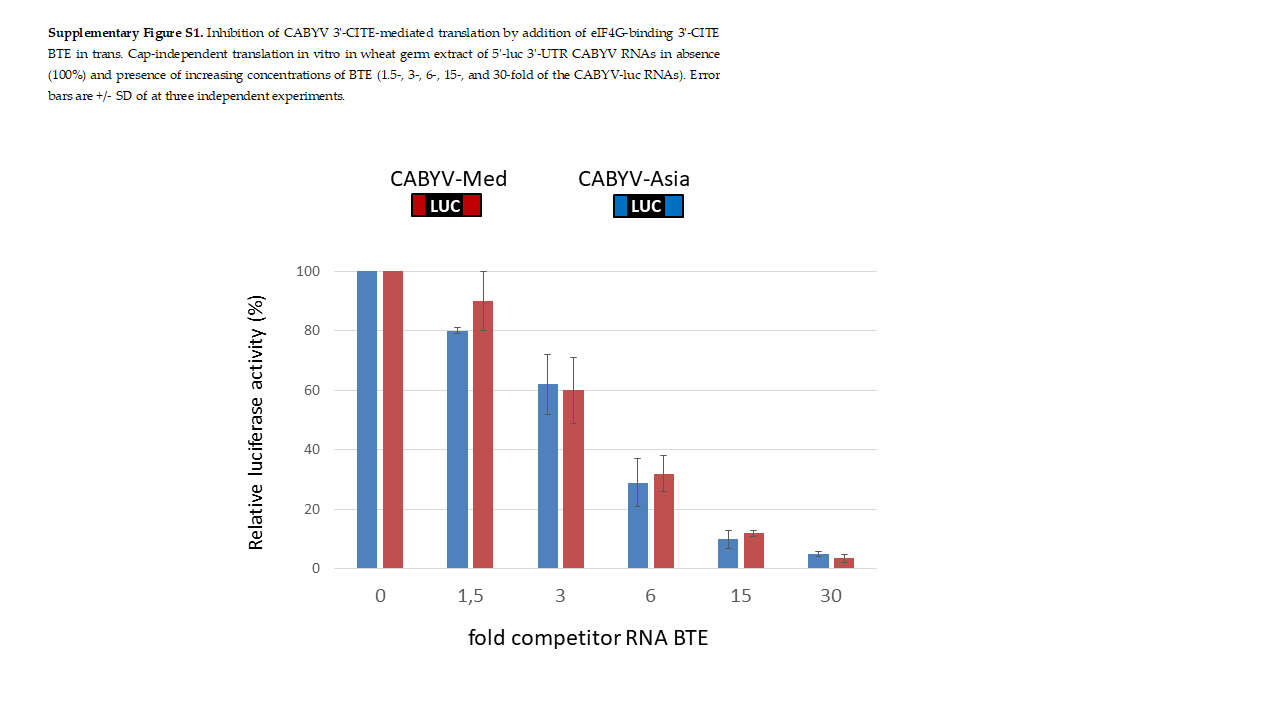

Supplement: Supplementary file 1 [file ijms-24-17598-s001.zip › SupplFigureS1.tif]

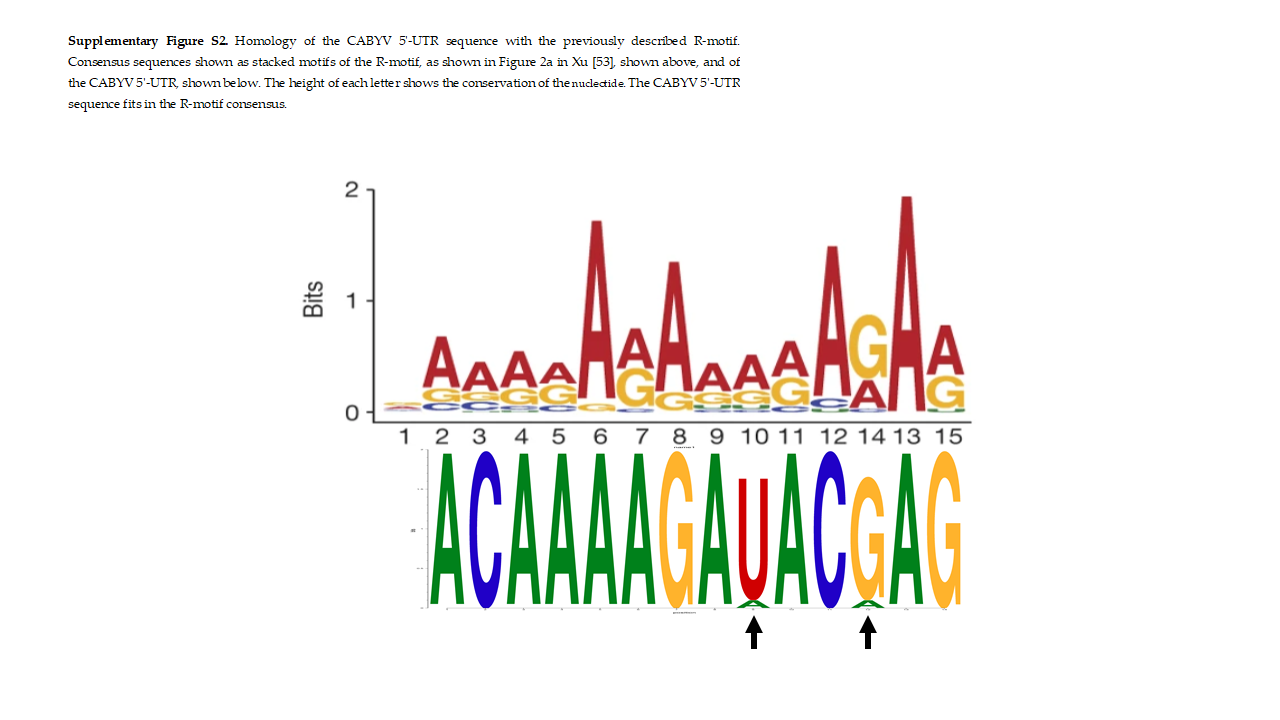

Supplement: Supplementary file 1 [file ijms-24-17598-s001.zip › SupplFigureS2.tif]
